# Analysis of influencing factors and a predictive model of small airway dysfunction in adults

**DOI:** 10.1186/s12890-023-02416-5

**Published:** 2023-04-25

**Authors:** Yifan Zhang, Haihua Zhang, Xuan Su, Ying Wang, Guizhou Gao, Xiaodong Wang, Tao Zhang

**Affiliations:** 1 Department of Thoracic Surgery, Tangdu Hospital, Air Force Medical University, Xi’an, 710032 China; 2 Department of Respiratory Medicine, Tangdu Hospital, Air Force Medical University, Xi’an, 710032 China

**Keywords:** Small airway, Pulmonary function test, Risk factors, Nomogram

## Abstract

**Background:**

Small airway dysfunction (SAD) is a widespread but less typical clinical manifestation of respiratory dysfunction. In lung diseases, SAD can have a higher-than-expected impact on lung function. The aim of this study was to explore risk factors for SAD and to establish a predictive model.

**Methods:**

We included 1233 patients in the pulmonary function room of TangDu Hospital from June 2021 to December 2021. We divided the subjects into a small airway disorder group and a non-small airway disorder group, and all participants completed a questionnaire. We performed univariate and multivariate analyses to identify the risk factors for SAD. Multivariate logistic regression was performed to construct the nomogram. The performance of the nomogram was assessed and validated by the Area under roc curve (AUC), calibration curves, and Decision curve analysis (DCA).

**Results:**

One. The risk factors for small airway disorder were advanced age (OR = 7.772,95% CI 2.284–26.443), female sex (OR = 1.545,95% CI 1.103–2.164), family history of respiratory disease (OR = 1.508,95% CI 1.069–2.126), history of occupational dust exposure (OR = 1.723,95% CI 1.177–2.521), history of smoking (OR = 1.732,95% CI 1.231–2.436), history of pet exposure (OR = 1.499,95% CI 1.065–2.110), exposure to O_3_ (OR = 1.008,95% CI 1.003–1.013), chronic bronchitis (OR = 1.947,95% CI 1.376–2.753), emphysema (OR = 2.190,95% CI 1.355–3.539) and asthma (OR = 7.287,95% CI 3.546–14.973). 2. The AUCs of the nomogram were 0.691 in the training set and 0.716 in the validation set. Both nomograms demonstrated favourable clinical consistency. 3.There was a dose‒response relationship between cigarette smoking and SAD; however, quitting smoking did not reduce the risk of SAD.

**Conclusion:**

Small airway disorders are associated with age, sex, family history of respiratory disease, occupational dust exposure, smoking history, history of pet exposure, exposure to O_3_, chronic bronchitis, emphysema, and asthma. The nomogram based on the above results can effectively used in the preliminary risk prediction.

## Introduction

A small airway is usually defined as one with a lumen diameter less than 2 mm [1]. This area is called the quiet zone of the lung because it is difficult to detect with existing instruments [2]. However, respiratory diseases are often caused by pathological changes in the small airways. Airway dysfunction can be a precursor to lung disease [3], and in advanced lung disease, small airway obstruction can severely impact lung function [4, 5]. Among patients with chronic respiratory disease, patients with small airway dysfunction (SAD) are more prone to wheezing or sputum production [6]. A previous study found that patients with SAD who require thoracotomy for pulmonary nodules are more likely to develop postoperative inflammation and emphysema than patients with normal lung function [7]. For people with asthma, the dysfunction caused by persistent inflammation of the peripheral small airway is closely related to the degree of asthma control [8]. Therefore, the prevention of small airway dysfunction is of great significance to human health.

Measurements of small airway function can be used to screen people with early-stage lung disease or people who are at risk for lung disease; however, while the inaccessibility of small airways has made it difficult to identify early physiological abnormalities, recent advances in medical technology have yielded many methods for assessing small airway function [9]. Spirometry is a method for diagnosing airflow limitation that requires patient cooperation [10]. There are three measures of lung function that we can use to assess SAD: maximal mid-expiratory flow (MMEF), forced expiratory flow 50% (FEF_50%_), and forced expiratory flow 75% (FEF_75%_). SAD is diagnosed when at least two of these three measures are below 65% of the predicted value [11]. Spirometry can more accurately detect SAD in patients than forced expiratory volume in 1 s (FEV_1_) [12].

Several Western studies have reported the prevalence and influencing factors of SAD. These studies used spirometry with different diagnostic criteria and selected populations that were largely specific and did not represent the general population. In addition, the reported prevalence varies widely, ranging from 6.7% in veterans to 53.8% in people with asthma. The most representative study in China is a report on SAD by the China Lung Health Research Group (CPH), which found that the prevalence reached 57.7%. Approximately 426 million adults nationwide suffer from SAD. Therefore, the prevention of small airway disease deserves attention [13].

Small airway function is affected by a variety of factors, and a range of lifestyle and health conditions may also be associated with small airway function. Exposure to ambient air pollutants may cause particles to enter the circulation through the capillary bed and accumulate in the alveoli, leading to a short-term decline in lung function; furthermore, long-term alcohol use reduces the phagocytic function of macrophages and leads to inflammation, which causes a decrease in lung function [14]. In a Korean study, the incidence of chronic obstructive pulmonary disease decreased from 14.1% to 5.9% as the frequency of green tea intake increased, and the frequency of green tea intake was linearly related to FEV1/FVC [15]. The endurance and strength of the respiratory muscles reduce the systemic inflammatory response and can effectively improve lung function [16]. A cross-sectional study in the United States showed that pet feeding may increase airway inversion, thereby increasing the likelihood of asthma [17]. Another study found a nonlinear relationship between diabetes mellitus and pulmonary function as well as a significant correlation between diabetes mellitus and the decrease in FEV1 and FVC [18]. A Japanese study found that decreased respiratory function was associated with increased ambulatory blood pressure, especially during the day [19]. It is worthwhile to research whether the changes in lung function of these factors are related to SAD. The aim of this study was to investigate the risk factors for SAD, to determine the relationship between these risk factors and small airway function, and to develop and validate a risk prediction model for the screening of SAD.

## Materials and methods

### Study design and participants

This cross-sectional survey was conducted in the Pulmonary Function Room of the Second Affiliated Hospital of Air Force Military Medical University. We continuously recruited adults 18 and older who completed lung function tests between June 2021 and December 2021. All participants completed a standard respiratory epidemiological questionnaire. The exclusion criteria were as follows: breast, abdominal, or eye surgery in the past 3 months; retinal detachment or myocardial infarction for which spirometry cannot be performed; heart rate exceeding 120 beats per minute; hospitalization for chronic obstructive pulmonary disease deterioration in the last 4 weeks; active TB or antibacterial chemotherapy for newly discovered tumours; or current pregnancy or breastfeeding. The Ethics Committee of the Second Affiliated Hospital of the Air Force Medical University approved the study protocol, and all subjects participated in the study.

### Questionnaire

The sociodemographic questionnaire assessed education status, family history, BMI (body mass index, kg/m^2^), dust exposure history, smoking (smokers are those who reported ever smoking, smoking ≥ 100 cigarettes within 1 year, or smoking at least weekly 2 cigarettes for more than 1 year in a row), exercise habits (active exercise was indicated by every exercise session being < 30 min, 30–60 min, or > 60 min more than 3 times a week or having a total duration of physical activity exceeding 60 min per week), green tea consumption, alcohol use, pet ownership (i.e., pets or livestock with fur, such as cats, dogs, cows, or sheep), history of respiratory disease [20], history of hypertension, and history of diabetes. We also recorded daily levels of air pollutants, including PM_2.5_ (μg/m^3^), PM_10_ (μg/m^3^), SO_2_ (μg/m^3^), CO (mg/m^3^), NO_2_ (μg/m^3^), O_3_ (μg/m^3^) and the Air Quality Index (AQI); these data were collected from the China National Environmental Monitoring Centre (CNEMC).

### Pulmonary function test

Pulmonary function was assessed by trained technicians using the Jaeger Master Screen PFT System spirometer [21]. All participants had their lung function tested. The participant was asked to perform up to eight forced expiratory movements until FVC and FEV1 are repeatable within 150 ml. All spirometry data were centrally reviewed by an expert panel based on American Thoracic Society and European Respiratory Society standards, and spirometry reference values and low-quality data were excluded [22]. According to the South diagnostic criteria, if two of the three measures (i.e., MMEF, MEF50%, and MEF25%) are lower than 65% of the predicted value, the patient will be diagnosed with SAD [11].

### Statistical analysis

Using SPSS 26.0 statistical software, each factor was analysed by univariate logistic regression analysis. Multivariate logistic regression analysis was performed to assess the risk factors for small airway disorder and to calculate odds ratios (OR). The models were adjusted for age, sex, family history of respiratory disease, occupational dust exposure, smoking history, history of pet exposure, exposure to O3, chronic bronchitis, emphysema, and asthma. *P* < 0.05 was considered statistically significant. An OR > 1.0 was considered to indicate a risk factor for the occurrence of SAD, while an OR < 1.0 was considered to indicate a protective (preventive) factor against the occurrence of SAD.

For the construction and validation of the nomogram, the subjects were randomly divided into a training set and a validation set at a ratio of 2:1. Following the multivariate analysis, factors with a two-sided p value < 0.05 were selected to construct the nomograms. The predictive accuracy of the nomograms was measured by the Area under roc curve (AUC) of the Receiver operating characteristic (ROC) curve in both the training and validation sets. The consistency between the actual outcomes and predicted probabilities was measured by the calibration curve. The clinical utility of the nomograms was measured by Decision curve analysis (DCA) and clinical impact curves for a sample size of 1000.

## Results

**Fig. 1:**
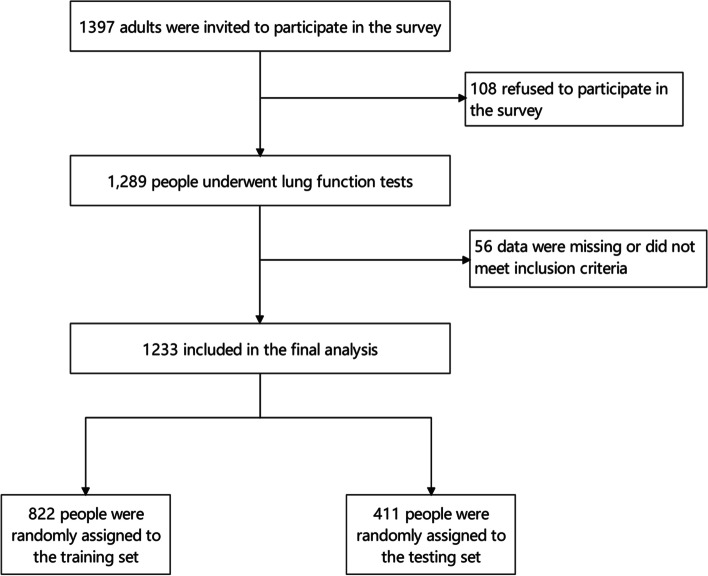
Flow of participants through the study

Of the 1397 participating adults, 1289 underwent the pulmonary function tests and completed the questionnaire (92.1% response rate), of which 56 were excluded due to missing data or not meeting the inclusion criteria. Thus, 1233 participants were included in this study (822 in the training set and 411 in the validation set) (see Fig. 1). In our cohort, the mean age of the participants was 52.97 years, and 55.15% (680 of 1233) of the study population had SAD. The older the person was, the higher the probability of developing SAD (Table 1). The prevalence of SAD increased from 38.46% (30/78) in 18–29-year-olds to 81.8% in individuals aged 80 years and older (18/22). The prevalence of SAD was 61.1% in smokers and 51.2% in never-smokers (*P* = 0.001). In addition, education level (*P* < 0.001), dust exposure history (*P* = 0.004), family history of respiratory disease (*P* < 0.001), pet feeding status (*P* = 0.012), history of chronic bronchitis (*P* < 0.001), history of emphysema (*P* < 0.001), asthma (*P* < 0.001) (see Table 1) and O_3_ (μg/m^3^) (*P* = 0.002) (see Table 2) were significant factors in the univariate analysis.

**Table 1:** Single factor analysis of SAD

	total(*n* = 1233)	Non-SAD	SAD	*p* Value
		*n* = 553	*n* = 680	
age(years)	52.97 ± 14.1	50 ± 13.9	55.21 ± 13.8	< 0.001^***^
Age group (years)				< 0.001^***^
18–29	78(6.3%)	48(8.7%)	30(4.4%)	
30–39	164(13.3%)	90(16.3%)	74(10.9%)	
40–49	230(18.7%)	113(20.4%)	117(17.2%)	
50–59	328(26.6%)	152(27.5%)	176(25.9%)	
60–69	289(23.4%)	106(19.2%)	183(26.9%)	
70–79	122(9.9%)	40(7.2%)	82(12.1%)	
80 +	22(1.8%)	4(0.7%)	18(2.6%)	
Gender				0.275
male	650(52.7%)	282(51.0%)	368(54.1%)	
female	583(47.3%)	271(49.0%)	312(45.9%)	
BMI				0.695
< 18.5	44(3.6%)	17(3.1%)	27(4.0%)	
18.5–24.9	550(44.6%)	249(45.0%)	301(44.3%)	
> 25	639(51.8%)	287(51.9%)	352(51.8%)	
Educational Status				< 0.001^***^
Primary school or less	669(54.3%)	278(50.3%)	391(57.5%)	
Middle and high school	228(18.5%)	91(16.5%)	137(20.1%)	
College and higher	336(27.3%)	184(33.3%)	152(22.4%)	
History of dust exposure				0.004^**^
No	1073(87.0%)	498(90.1%)	575(84.6%)	
Yes	160(13.0%)	55(9.9%)	105(15.4%)	
Family history				< 0.001^***^
No	1030(83.5%)	486(87.9%)	544(80.0%)	
Yes	203(16.5%)	67(12.1%)	136(20.0%)	
Currently smoking				0.001^**^
No	742(60.2%)	362(65.5%)	380(55.9%)	
Yes	491(39.8%)	191(34.5%)	300(44.1%)	
Passive smoking				0.114
No	748(60.7%)	322(58.2%)	422(62.6%)	
Yes	485(39.3%)	231(41.8%)	254(37.4%)	
Biomass fuel				0.084
No	1025(83.1%)	471(85.2%)	554(81.5%)	
Yes	208(16.9%)	82(14.8%)	126(18.5%)	
Green tea				0.081
Never	914(74.1%)	400(72.3%)	514(75.6%)	
Occasionally	103(8.4%)	57(10.3%)	46(6.8%)	
Always	216(17.5%)	96(17.4%)	120(17.6%)	
Drinking				0.946
Never	1041(84.4%)	469(84.8%)	572(84.1%)	
Occasionally	71(5.8%)	31(5.6%)	40(5.9%)	
Always	121(9.8%)	53(9.6%)	68(10.0%)	
Sporting				0.907
No	709(57.5%)	319(57.7%)	390(57.4%)	
Yes	524(42.5%)	234(42.3%)	290(42.6%)	
Pet Keeping				0.012*
No	1021(82.8%)	473(85.5%)	548(80.6%)	
< 1 Year	23(1.9%)	13(2.4%)	10(1.5%)	
> 1Year	189(15.3%)	67(12.1%)	122(17.9%)	
History of chronic bronchitis				< 0.001^***^
No	1009(81.8%)	494(89.3%)	515(75.7%)	
Yes	224(18.2%)	59(10.7%)	165(24.3%)	
History of emphysema				< 0.001^***^
No	1104(89.5%)	526(95.1%)	578(85.0%)	
Yes	129(10.5%)	27(4.9%)	102(15.0%)	
History of asthma				< 0.001^***^
No	1159(94.0%)	543(98.2%)	616(90.6%)	
Yes	74(6.0%)	10(1.8%)	64(9.4%)	
Hypertension				0.099
No	975(79.1%)	449(81.2%)	526(77.4%)	
Yes	258(20.9%)	104(18.8%)	154(22.6%)	
Diabetes				0.192
No	1125(91.2%)	511(92.4%)	614(90.3%)	
Yes	108(8.8%)	42(7.6%)	66(9.7%)	

Data are n (%), n/N (%). The differing denominators used in the calculation of percentages are because of missing data. Small airway dysfunction was defined as at least two of maximal mid-expiratory flow, forced expiratory flow (FEF) 50% and FEF 75% having below 65% of the predicted values

^*^P < 0.05

^**^P < 0.01

^***^P < 0.001

**Table 2:** Air pollutants and SAD K-S rank sum test

	Non-SAD	SAD	*z* Value	*p* Value
O_3_(ug/m^3^)	645.62(ug/m^3^)	581.81(ug/m^3^)	-3.133	0.002^**^
SO_2_(ug/m^3^)	602.46(ug/m^3^)	634.88(ug/m^3^)	-1.852	0.064
CO (mg/m^3^)	601.53(mg/m^3^)	636.02(mg/m^3^)	-1.721	0.085
NO_2_(ug/m^3^)	604.43(ug/m^3^)	632.46(ug/m^3^)	-1.377	0.169
PM_2.5_(ug/m^3^)	606.01(ug/m^3^)	630.51(ug/m^3^)	-1.205	0.228
PM_10_(ug/m^3^)	619.58(ug/m^3^)	613.83(ug/m^3^)	-0.282	0.778
AQI	615.04	619.41	-0.215	0.83

*PM2·5* Particulate matter with a diameter less than 2·5 µm, *PM10* Particulate matter with a diameter less than 10 µm, *O3* Ozone, *SO2* Sulfur dioxide, *CO* Carbon monoxide, *NO2* Nitrogen dioxide, *AQI* Air Quality Index

^*^P < 0.05

^**^P < 0.01

^***^P < 0.001

However, sex (*P* = 0.275), BMI (*P* = 0.695), passive smoking (*P* = 0.114), biomass fuel use (*P* = 0.084), green tea consumption (*P* = 0.081), alcohol use (*P* = 0.946), exercise (*P* = 0.907), hypertension (*P* = 0.099), diabetes (*P* = 0.192), PM2.5 (μg/m^3^) (*P* = 0.228), PM10 (μg/m^3^) (*P* = 0.778), SO2 (μg/m^3^) (P = 0.064), CO (mg/m^3^) (P = 0.085), NO2 (μg/m^3^) (*P* = 0.169), and the AQI (P = 0.830) were not significant factors for SAD in the univariate analysis.

**Fig. 2:**
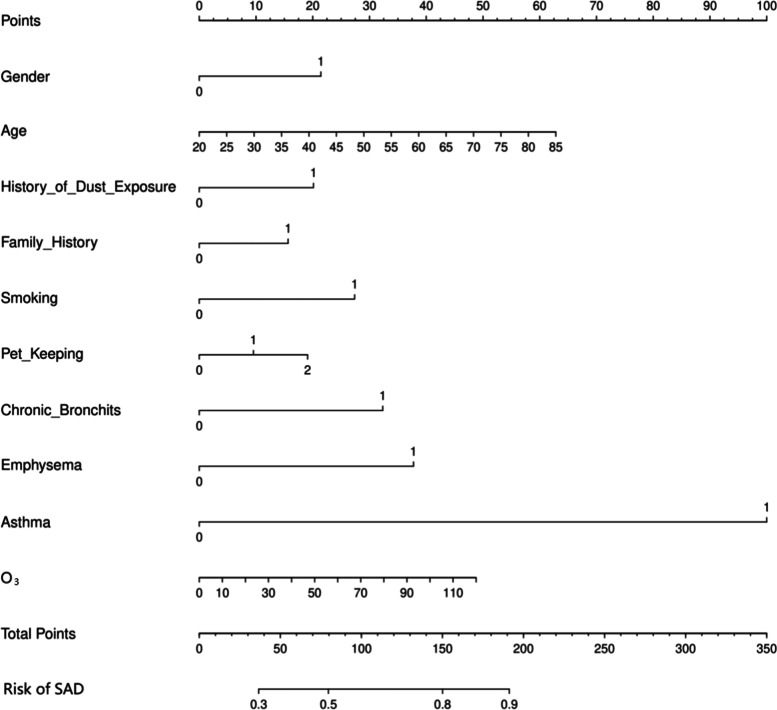
Nomogram for the prediction of SAD. Nomogram was constructed based on the data of logistic analysis. The points of each feature were added to obtain the total points, and a vertical line was drawn on the total points to obtain the corresponding ‘risk of SAD’. SAD: small airway dysfunction.

**Fig. 3:**
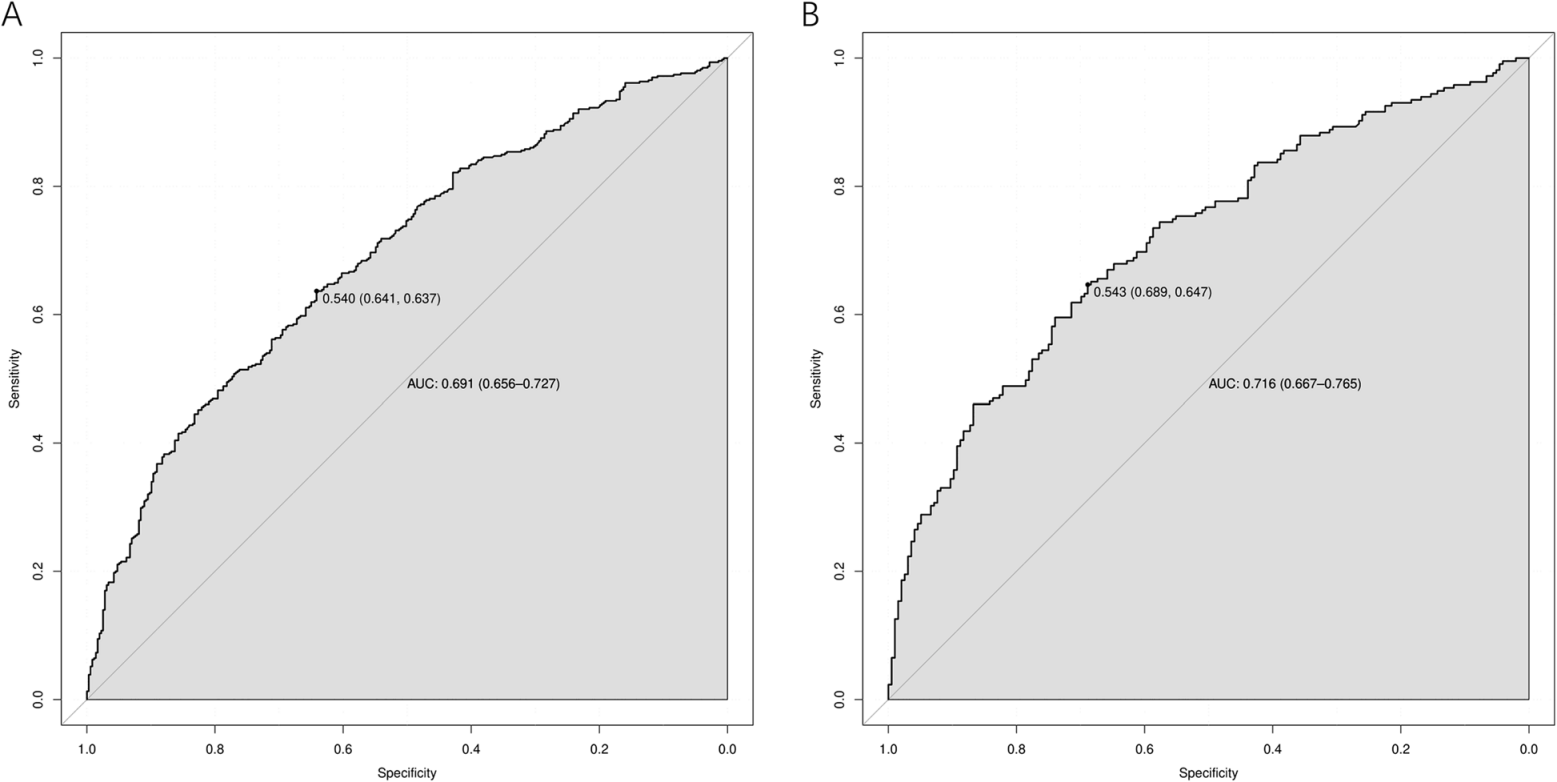
ROC curves for the prediction of SAD in the training set and validation set. **A**: ROC curves of the factors and nomogram in the training set. **B**: ROC curves of the factors and nomogram in the validation set. ROC: Receiver operating characteristic; SAD: small airway dysfunction

**Fig. 4:**
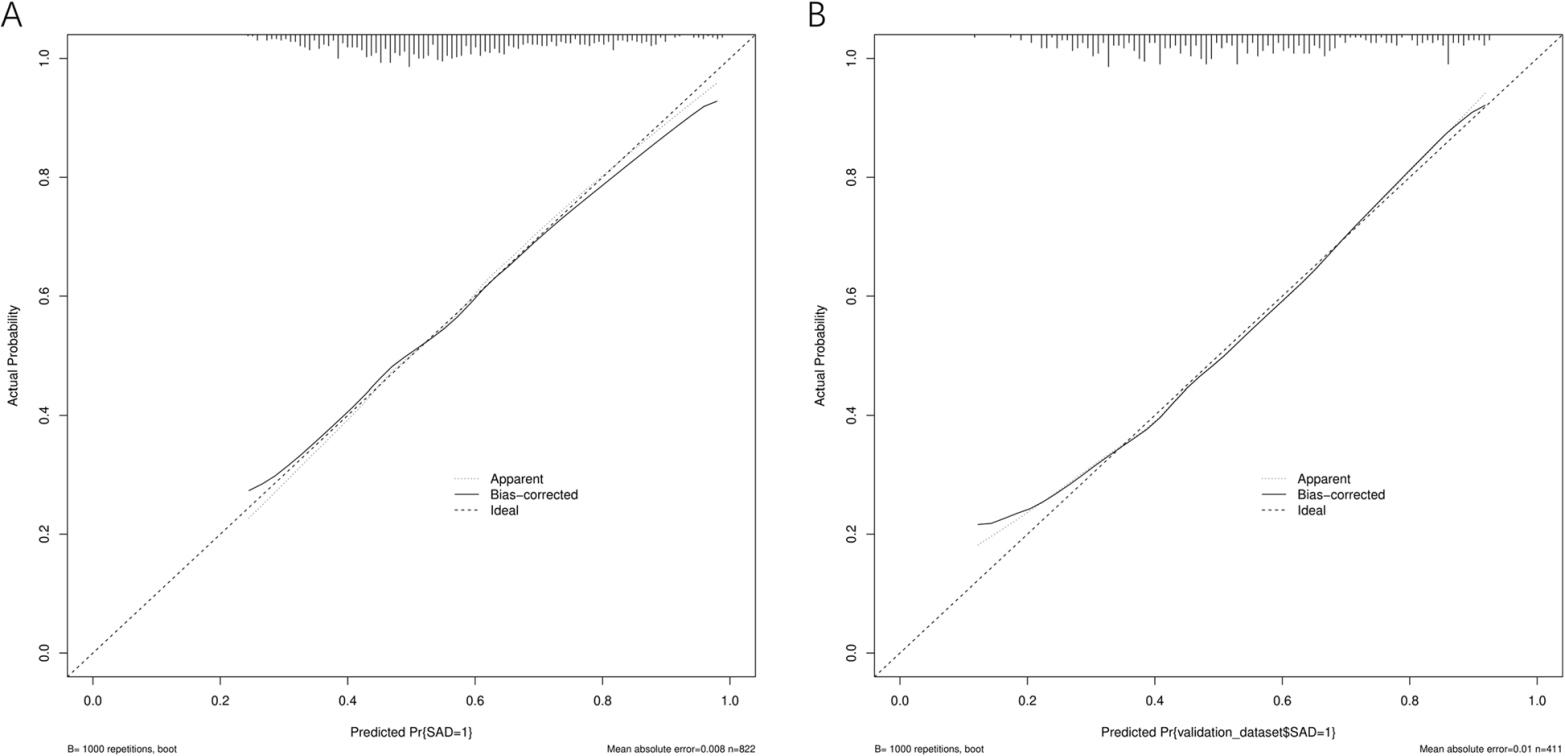
Calibration curves of nomogram prediction in the training set and validation set. **A**: Calibration curves of nomogram prediction in the training set. **B**: Calibration curves of nomogram prediction in the validation set

**Fig. 5:**
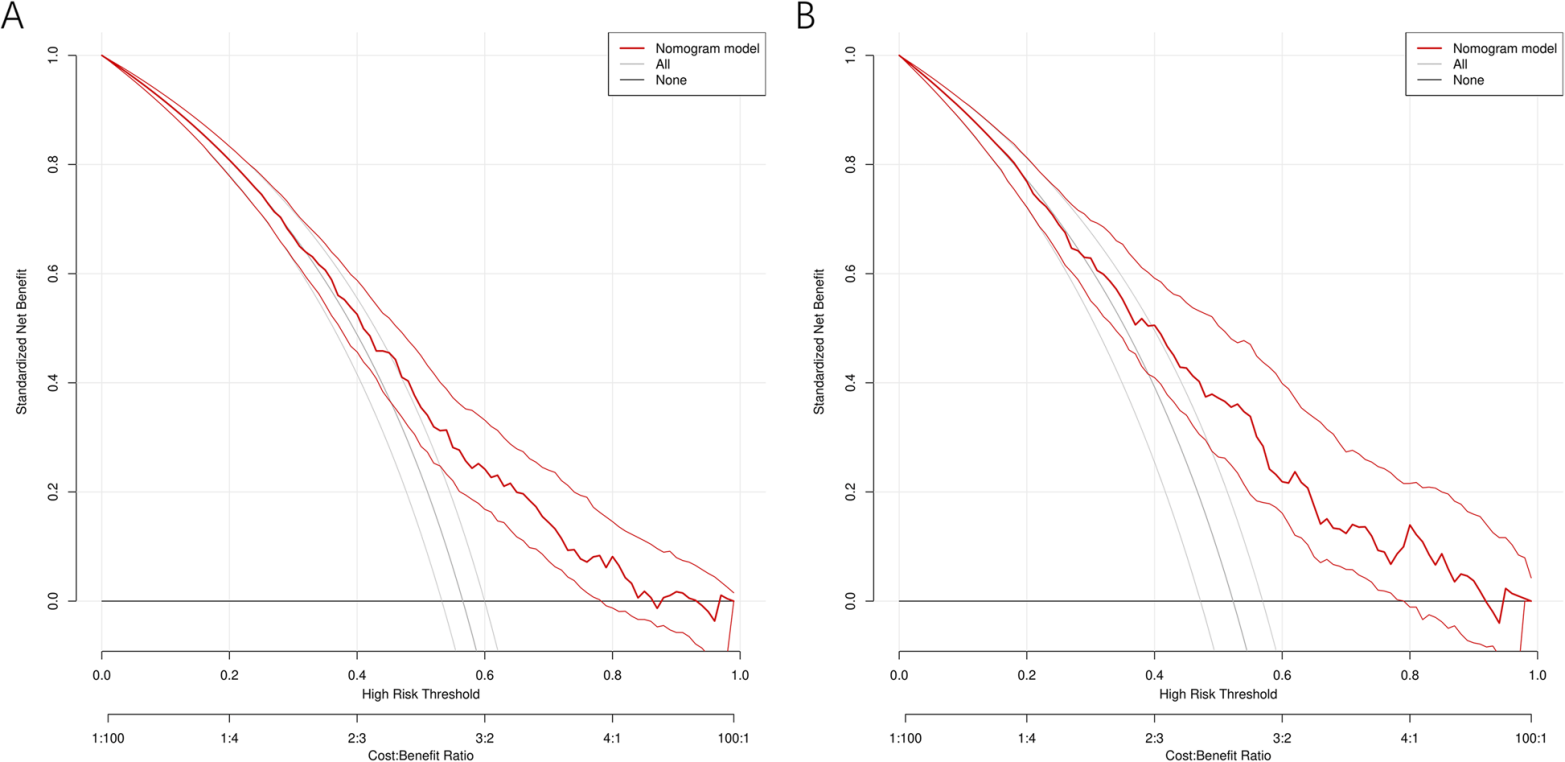
DCA of nomogram prediction in the training set and validation set. **A**: DCA of nomogram prediction in the training set. **B**: DCA of nomogram prediction in the validation set. DCA: Decision curve analysis

Multivariate analysis revealed that advanced age (OR = 7.772, 95% CI 2.284–26.443), female sex (OR = 1.545 95% CI 1.103–2.164), family history of respiratory disease (OR = 1.508 95% CI 1.069–2.126), history of occupational dust exposure (OR = 1.723 95% CI 1.177–2.521), history of smoking (OR = 1.732 95% CI 1.231–2.436), history of pet exposure (OR = 1.499, 95% CI 1.065–2.110), exposure to O3 (OR = 1.008 95% CI 1.003–1.013), chronic bronchitis (OR = 1.947 95% CI 1.376–2.753), emphysema (OR = 2.190 95% CI 1.355–3.539) and asthma (OR = 7.287 95% CI 3.546–14.973) (see Table 3) were significant influencing factors of SAD. These 10 independent factors were used to construct the nomogram (Fig. 2), and the performance of the nomogram was assessed with the area under the receiver operating characteristic curve (AUC). The AUC value of the training set was 0.691 (95% CI: 0.656–0.727), and the AUC value of the validation set was 0.716 (95% CI: 0.667–0.765) (Fig. 3), thus indicating that the model had good predictive discrimination. Furthermore, the calibration curve showed a high consistency between the prediction and actual observation (Fig. 4). The accuracy of the SAD risk prediction model was evaluated by using the standardized net benefit as the longitudinal coordinate and the high-risk threshold as the horizontal coordinate. The DCA curve was drawn (Fig. 5), and the SAD risk prediction model was used to predict the net benefit rate of SAD incidence, which was always > 0 and had clinical significance.

**Table 3:** Risk factors for small airway disorders

	Case(n)	*OR* (95% CI)	*p* Value
Age group (years)			
18–29	30	1	
30–39	74	1.387(0.770–2.500)	0.276
40–49	117	1.693(0.964–2.973)	0.067
50–59	176	1.83(1.060–3.159)	0.03^*^
60–69	183	2.606(1.491–4.557)	0.001^**^
70–79	82	3.001(1.577–5.711)	0.001^**^
80 +	18	7.772(2.284–26.443)	0.001^**^
Gender			
Male	368	1	0.011^*^
Female	312	1.545(1.103–2.164)	
Currently smoking			
No	380	1	0.002^**^
Yes	300	1.732(1.231–2.436)	
History of dust exposure			
No	575	1	0.005^**^
Yes	105	1.723(1.177–2.521)	
Pet Keeping			
never	548	1	
< 1 year	10	0.498(0.205–1.208)	0.123
> 1 year	122	1.499(1.065–2.110)	0.02^*^
Family history			
No	544	1	0.024^*^
Yes	136	1.481(1.052–2.084)	
O3(ug/m^3^)		1.008(1.003–1.013)	0.001^**^
History of chronic bronchitis			
No	515	1	< 0.001^***^
Yes	165	1.947(1.376–2.753)	
History of emphysema			
No	578	1	0.001^**^
Yes	102	2.19(1.355–3.539)	
History of asthma			
No	616	1	< 0.001^***^
Yes	64	7.287(3.546–14.973)	

adjusted for Model, the risk of SAD was associated with age, Gender,dust exposure history, family history, smoking, pet ownership, O3(ug/m3), history of chronic bronchitis, history of emphysema and history of asthma were significantly associated. Small airway dysfunction was defined as at least two of maximal mid-expiratory flow, forced expiratory flow (FEF) 50% and FEF 75% having below 65% of the predicted values

^*^P < 0.05

^**^P < 0.01

^***^P < 0.001

**Fig. 6:**
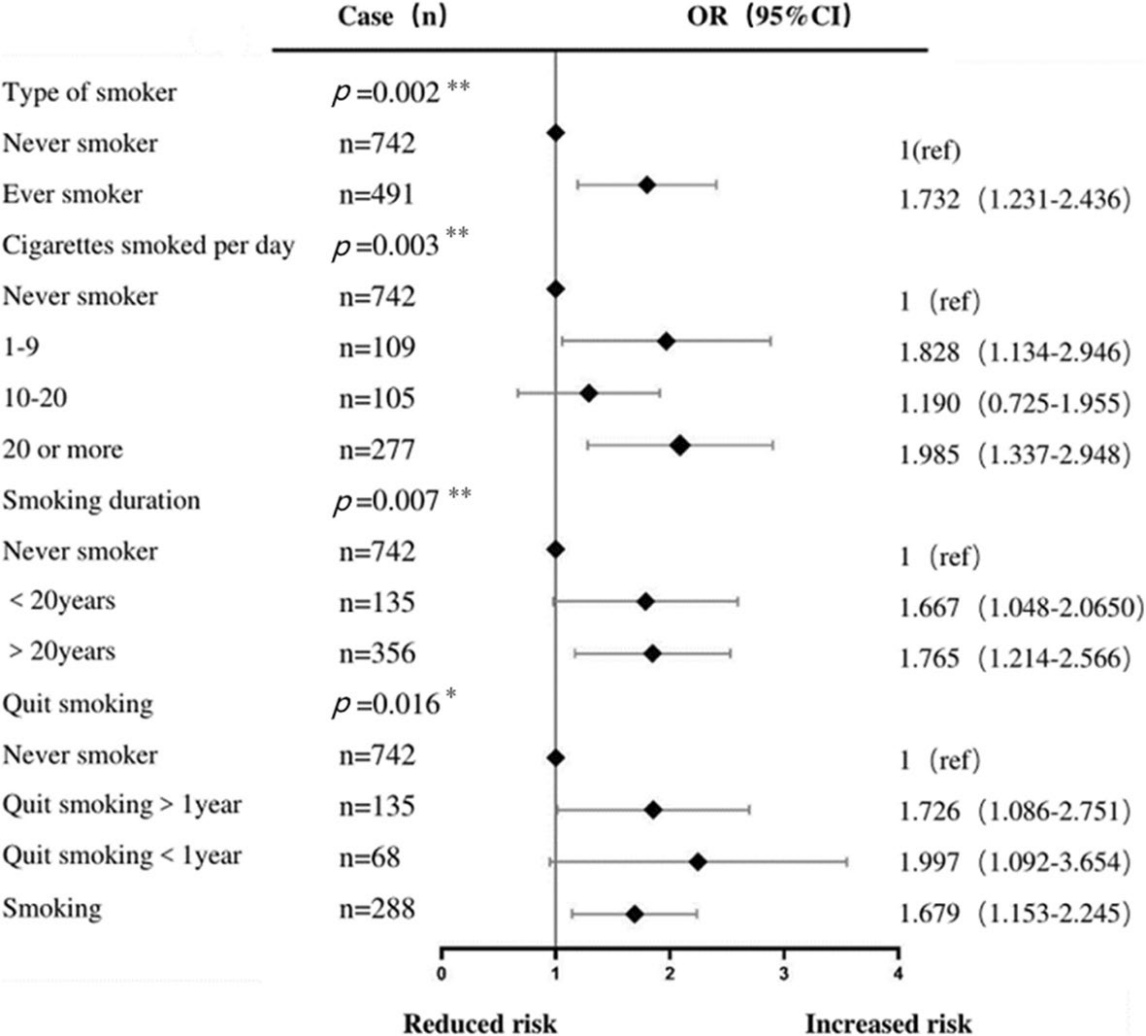
Effects of daily smoking, smoking duration, and smoking cessation on Small Airway function Each Point represents an OR. The horizontal lines indicate 95% CIs. The x-axis was based on log scale. ORs are adjusted for age, dust exposure history, family history, smoking, pet ownership, O_3_(ug/m^3^), history of chronic bronchitis, history of emphysema and history of asthma. OR:odds ratio. O_3_: ozone

Among the preventive factors, we further analysed patients' smoking status and pet ownership. We analysed the smoking group and found a dose‒response relationship between cigarette smoking and SAD; however, quitting smoking did not reduce the risk of SAD (Fig. 6). We found that SAD was related to the number of years a pet was kept (*P* = 0.039) but not the type of pet (*P* = 0.467).

## Discussion

SAD is an age-related disease (OR = 7.772, 95% CI 2.284–26.443) that is more common in elderly individuals [13, 23]. Changes in the network of curled collagen fibres surrounding the alveolar duct and adjacent alveoli lead to dilatation of the alveolar duct and expansion of the alveolar space, which in turn leads to alveolar enlargement. The result is a decrease in alveolar surface tension, leading to a decrease in alveolar compliance. Furthermore, an increase in age is associated with a decrease in the vertebral body and an increase in the convexity of the thorax, thus resulting in an increase in chest diameter. In addition, changes in the chest wall result in a decrease in the curvature of the diaphragm, and some extrathoracic causes lead to decreased respiratory muscle mass and reduced airway function [24].

Small airway disorders were found to be gender-related, such that women had a higher risk of SAD than men (OR = 1.545 95% CI 1.103–2.164). This finding was consistent with previous studies [25]. A study in a mouse chronic obstructive pulmonary disease model showed that compared with male mice, chronic smoke exposure increased the risk of airway remodelling in female mice, which could be prevented by removing the ovaries. It was suggested that oxidative stress, increased TGF-β1 signal transduction and the effect of oestrogen were responsible for this phenomenon [26].

We analysed the effect of family history of respiratory disease on SAD (OR = 1.481 95% CI 1.052–2.084); the results were consistent with the results of Okyere DO [27]. Family history of interstitial lung disease, COPD, and asthma have been studied as factors for SAD [28–30]. Genetic analyses revealed that in the Maas and ALSPAC species, 77 single-nucleotide polymorphisms were found to be associated with FEV1/FVC or FEV1 decreases [31].

Dust exposure history (OR = 1.723 95% CI 1.177–2.521) was also one of the risk factors for small airway obstruction. A 15-year follow-up of 9/11 survivors exposed to high concentrations of dust showed that they had higher airway responsiveness than the general population [32]. Vasanthi R Sunil conducted research on this dust component in mice and found that dust exposure touch history leads to lung inflammation and oxidative stress and is related to changes in lung epigenetics and pulmonary dynamics [33]. Multiple epidemiological studies have also verified the negative impact of dust exposure history on lung function [34–36].

Smoking is the most important preventable risk factor for SAD. Our study found that smoking is associated with SAD (OR = 1.732 95% CI 1.231–2.436). Studies have shown that smoking and lung ageing metabolism are the main causes of chronic obstructive pulmonary disease emphysema development, as demonstrated in a previous mouse model [37]. Another pair of studies showed that in male subjects, smoking caused abnormal expression of several ageing-related genes in small airway epithelial cells. Among smokers, the length of telomeres in small airway epithelial cells was significantly reduced by 14% compared with nonsmokers [38]. Chronic smoking can lead to inflammation, injury, tissue remodelling, and eventually airway dysfunction, which leads to inhibited airflow and impaired alveolar ventilation [39]. Standardized smoking rates are reported to be high in China: the prevalence of current smoking is 26.0% (95% CI 25.8–26.2), and the standardized smoking rate for women under 40 years of age increased from 1.0% in 2003 to 1.6% in 2013. In addition, the prevalence of smoking among individuals aged 15–24 increased from 8.3% in 2003 to 12.5% in 2013 [40]. In addition, in our study, we found a dose–response relationship between cigarette smoking and SAD, but we found no association between smoking cessation and SAD, this is slightly different from the results of other reports [13]. Our study demonstrates that the impairment of small airway function is irreversible regardless of whether you quit smoking. On the other hand, this conclusion may also be related to demographics differences in subjects [41], whose effects on small airways need to be observed in further cohort studies.

There is little evidence on the relationship between SAD and pet ownership. However, a study by Edith B Milanzi revealed that early childhood exposure to pets may slow the growth of FVC during adolescence [42], and this correlation may be related to Toxoplasma gondii [43]. Other studies have found that owning a cat is associated with a reduced risk of childhood asthma, while owning rabbits and rodents is associated with an increased risk of childhood asthma [44]. The NHANES study showed that both cats and dogs may be allergens related to the development of asthma [45]. In our study, small airway function was found to be associated with time spent with pets (OR = 1.499 95% CI 1.065–2.110). No significant differences were found between pet species (*P* = 0.32), and the mechanisms behind these differences deserve further study.

We also investigated the relationship between air pollutants and SAD. We recorded the air pollution level and found that the O_3_ level in the air was correlated with SAD (OR = 1.008 95% CI 1.003–1.013). This relationship may be due to oxidative stress caused by acute exposure to ozone, increases in nitric oxide (NO) and other reactive nitrogen species in the lungs, which are then modified to produce S-nitroso mercaptan (SNO), thereby changing protein function and acting on macrophages and ultimately leading to lung inflammation. However, SAD was not found to be significantly associated with other air pollutants, including PM_2.5_, PM_10_, SO_2_, CO, and NO_2_, which may be due to a lag effect [46]. Further research is needed to explore these correlations.

There was a strong link between SAD and asthma (OR = 7.287 95% CI 3.546–14.973). In a study by Postma Dirkje S, SAD was found to be a risk factor for asthma and was present at all levels of asthma [47], particularly in patients with severe asthma. However, another study identified asthma as a risk factor for small airway disorders [48]. One study contradicted the idea that inhaling allergic or nonallergic irritants causes inflammation or contraction of smooth muscles, which reduces their diameter and increases airway resistance, leading to SAD. SAD also accelerates the progression of asthma [49]. This mechanism can also be applied to chronic bronchitis (OR = 1.947 95% CI 1.376–2.753) and emphysema (OR = 2.190 95% CI 1.355–3.539). Chronic bronchitis is caused by goblet cell overproduction and secretion of excess mucus, which can lead to small airway lumen obstruction, epithelial remodelling, and airway surface changes in facial tension. Consequently, these changes can amplify airflow obstruction, which can lead to airway collapse, and SAD can exacerbate chronic bronchitis clinical manifestations [50]. Another study of emphysema in smokers showed that the occurrence of small airway obstruction was associated with the progression of emphysema. This may imply that airway dysfunction precedes lung function decline, and SAD may serve as an independent predictor of emphysema. Early identification and preventive treatment can limit the progression of emphysema [51].

We found no significant associations between SAD and tea consumption (*P* = 0.657), alcohol consumption (*P* = 0.855), exercise (*P* = 0.356), diabetes (*P* = 0.921), and hypertension (*P* = 0.952). The reasons may be as follows. 1. The research subjects are different. The subjects of this study were outpatients, and there is a certain inherent bias among such samples. 2. This study is a cross-sectional study that assesses many influencing factors but cannot determine causality. A larger sample size is needed to further explore the factors that are correlated with SAD. 3. The association between the above factors and lung function is overestimated, and further cohort studies are needed to observe the internal relationship between the above factors and SAD.

In this study, we developed and validated a predictive nomogram for SAD based on retrospective cohort studies of adults according to diagnostic criteria for SAD. The nomogram contains 10 parameters, including age, female sex, family history, occupational dust exposure, smoking, pet ownership, exposure to O_3_, chronic bronchitis, emphysema, and asthma. All parameters can be easily assessed via questionnaire. Therefore, this nomogram can be used for self-assessment without a physician's assistance and may be helpful in the early prevention of SAD.

Our study has the following advantages. First, this is the first large-scale cross-sectional study of SAD in Xi'an. Second, this study included all age groups over the age of 18 and explored a wide range of living habits, health conditions and air pollution.

However, our study has some limitations. First, our research subjects were patients who visited the pulmonary function room of the hospital. Although pulmonary function testing has become a routine examination, the bias of the population cannot be ignored. Second, most of the participants in this study were residents from Xi'an and surrounding towns, so the influence of regional differences on the function of the small airway has not been explored. This effect proved to be nonnegligible [52]; second, we lack longitudinal data to substantiate some claims, including interventions such as smoking cessation, regarding whether preventive measures truly affect the progression of small airway disorders.

## Conclusion

Risk factors for patients with small airway disorders are advanced age, female sex, family history, occupational dust exposure, smoking, pet ownership, exposure to O3, chronic bronchitis, emphysema, and asthma. People with these risk factors should take appropriate precautions to prevent SAD. The nomograms based on the above results can effectively used in the preliminary risk prediction.

## Data Availability

The [Analysis of influencing factors of small airway dysfunction in adults] data used to support the findings of this study were supplied by [Tao Zhang] under license and so cannot be made freely available. Requests for access to these data should be made to [Tao Zhang, zhangft@fmmu.edu.cn].

## Abbreviations

SAD: Small airway dysfunction
MMEF: Maximal mid-expiratory flow
FEF_50%_: Forced expiratory flow 50%
FEF_75%_: Forced expiratory flow 75%
PM_2·5_: Particulate matter with a diameter less than 2·5 µm
PM_10_: Particulate matter with a diameter less than 10 µm
O_3_: Ozone
SO_2_: Sulfur dioxide
CO: Carbon monoxide
NO_2_: Nitrogen dioxide
AQI: Air Quality Index
ROC: Receiver operating characteristic
AUC: Area under roc curve
DCA: Decision curve analysis
